# Supplementary material for: Oribron: An Origami-Inspired Deformable Rigid Bronchoscope for Radial Support
Source: Micromachines (Basel). 2023 Apr 6;14(4):822. doi: 10.3390/mi14040822 (PMC10144517; doi:10.3390/mi14040822)
Supplement: Supplementary file 1 [file micromachines-14-00822-s001.zip › micromachines-2247065 - Supplementary File.pdf]

Supplementary Materials for

Oribron: An origami-inspired deformable rigid bronchoscope for radial support

Junjie Su [1], Yangyang Zhang [1], Liang Cheng [1], Ling Zhu [3], Runhuai Yang [1,*], Fuzhou Niu [2,*], Ke Yang [3,*] and Yuping Duan [1,*]

[1] School of Biomedical Engineering, Anhui Medical University, Hefei 230009, China; 332927506@qq.com (J.S.); yaungchang@foxmail.com (Y.Z.); 1596185478@qq.com (L.C.)

[2] School of Mechanical Engineering, Suzhou University of Science and Technology, Suzhou 215009, China

[3] Anhui Institute of Optics and Fine Mechanics, Hefei Institutes of Physical Science, Chinese Academy of Sciences, Hefei 230031, China; zhul@aiofm.ac.cn (L.Z.)

* Correspondence: yangrunhuai@ahmu.edu.cn (R.Y.); fzniu@usts.edu.cn (F.N.); keyang@aiofm.ac.cn (K.Y.); duanyuping126@126.com (Y.D.)

The PDF file includes:

Figure S1. Fabrication of Waterbomb.
Figure S2. Small size sample of Waterbomb.
Figure S3. The Waterbomb kinematic algorithm.
Figure S4. Complete CPs of Waterbomb.
Figure S5. Kinematic analysis of Waterbomb.
Figure S6. Geometric conflict.
Table. S1. Comparison of various methods to improve the utilization rate of oxygen.
Table. S2. Laser processing parameters.
Table. S3. Stability judgment of Waterbomb deformation process.
Table. S4. Stability judgment of various Waterbomb configurations.

Other Supplementary Material for this manuscript includes the following:

Video S1 (.mp4 format). Pneumatic control of Waterbomb.
Video S2 (.mp4 format). Smoke blocking of Waterbomb.

1 Research status of oxygen utilization rate

During the use of bronchoscope, there are mainly the following methods or devices can improve the utilization rate of oxygen: High-flow oxygen therapy, Oxygen humidifier, Oxygen mixer, Oxygen delivery device, Pulmonary expansion agent. We compared Oribron with a variety of methods, and the results are shown in the Table S1 below.

As can be seen from Table S1, the advantage of existing research is that it can achieve improved oxygen utilization and easily control the amount of oxygen required by the patient. However, on the one hand, they all require additional support devices, which will be increase the cost of treatment. On the other hand, these devices often lack some compatibility, and can't be adapted to a variety of patients. In this work, Oribron have many advantages, such as low cost (made from films), not required to connect other complex devices, and more modification and compatibility (origami structure is scale independent). Therefore, Oribron will provide a new strategy for the improvement of bronchoscope.

Table S1. Comparison of various methods to improve the utilization rate of oxygen.

**Methods or devices**	**Principle**	**Advantages**	**Disadvantages**
High-flow oxygen therapy [1-3]	By increasing the oxygen flow rate and concentration	(1) It can rapidly improve blood oxygen saturation.<br>(2) It is suitable for acute hypoxia patients.	(1) Additional devices is required.<br>(2) It may cause hyperoxiemia.
Oxygen humidifier [4-6]	Reduce irritation to respiratory mucosa by humidifying oxygen	Reduce the damage of oxygen to the trachea.	(1) Additional devices is required.<br>(2) It may cause bacterial contamination.
Oxygen mixer [7,8]	By mixing oxygen and air to provide different concentrations of oxygen	(1) It can provide different concentrations of oxygen.<br>(2) Reduce the damage of oxygen to the trachea.	(1) Additional devices is required.<br>(2) It may cause uneven concentration of oxygen.
Oxygen delivery device [9,10]	Deliver oxygen directly to the trachea	(1) It can provide different concentrations of oxygen.<br>(2) Reduce the damage of oxygen to the trachea.	(1) Additional devices is required.<br>(2) It may cause uneven concentration of oxygen.
Pulmonary expansion agent [11-13]	By dilating the alveoli to increase oxygen	(1) Improve the ventilation of the trachea.<br>(2) Reduce the resistance of the trachea.	(1) Additional devices is required.<br>(2) It may cause arrhythmia.
Oribron (this work)	Add Waterbomb structure to the bronchoscope body	(1) Low cost.<br>(2) No additional devices is required.<br>(3) Modifiable.	(1) High requirements for manual folding.<br>(2) High requirements for control stability.

2 Laser processing

The laser processing parameters of the three groups of samples were sorted out and plotted in Table S2. Depth and power are parameters set in the laser engraving software, and the specific values can be changed according to the thickness of thin films.

Table S2. Laser processing parameters.

Group	Thickness of materials (mm)	Depth (%)	Power (%)	Time (min)
i	PET (0.05)	1	1	85
ii	PET (0.05) + PI (0.03)	30	30	116
	PET (0.05) + PI (0.05)	50	50	118
iii	PET (0.05) + PTFE (0.03)	30	30	110
	PET (0.05) + PTFE (0.05)	50	50	115
Seal cap	PET (0.05)	1	1	8
Reinforced part	PET (0.20)	90	90	1

3 Fabrication of Waterbomb

The Waterbomb process consists of five steps, as shown in Figure S1.

Step1, Modify the Waterbomb CPs on the crease design software ORIPA (Ver.0.35) and transfer the crease pattern to the smartphone. To match the actual size (a = 14 mm), we set all crease widths (w) to 2 lb, resulting in a significant stiffness difference between the crease and facet.

Step 2, Set three key parameters of laser machining mode on phone: machining size, engraving depth (d) and power, see Table S2. Then, taking it connection with laser device through Bluetooth. In this study, all samples remained the same size, and the laser parameters (depth and power) were positively correlated with the thickness of the origami material. With the increase of depth and power, the processing time is extended, but the processing efficiency is higher (<2 hours).

Step 3, Use double-sided tape with thickness of 50 μm to bond the double layer films, and let PET film and laser contact directly. Because PET (top) has high stiffness, the film (bottom) with low stiffness can be retained after high temperature processing by laser, resulting in a significant stiffness difference between the creases (low stiffness) and the facets (high stiffness).

Step 4, Thin films are processed by laser device, aiming for the creases to produce the actual depth and width.

Step 5, Manually fold the finished film along the crease line, which takes about 10-15 minutes. It should be noted that the thickness of the seal cap and reinforced part is quite different, and the relative position relationship will change with the advance of the motion process. To ensure the structural stability of Waterbomb, we set the intersection line of the seal cap and reinforced part as a composite crease that can fold 360°.

As can be seen, the Waterbomb manufacturing process has the advantages of simplicity, low cost and machining precision. However, the manual folding error in step 5 makes the production process not strictly standardized, which can lead to the failure of the sample production when the size is small. Therefore, the engineering application of Waterbomb could be further expanded by incorporating machines with automatic folding behavior. In this work, we made a Waterbomb of minimum size a = 5 mm, as shown in Figure S2. Due to the great challenge of bonding the small size seal structure, the structure stability is particularly poor, as shown in Figure S2c. Therefore, subsequent pneumatic control cannot be achieved. However, we believe that with the continuous

improvement of processing technology, the small size of Waterbomb will have a wider application prospect, such as entering the human body as a microrobot to assist doctors to complete some difficult operations.

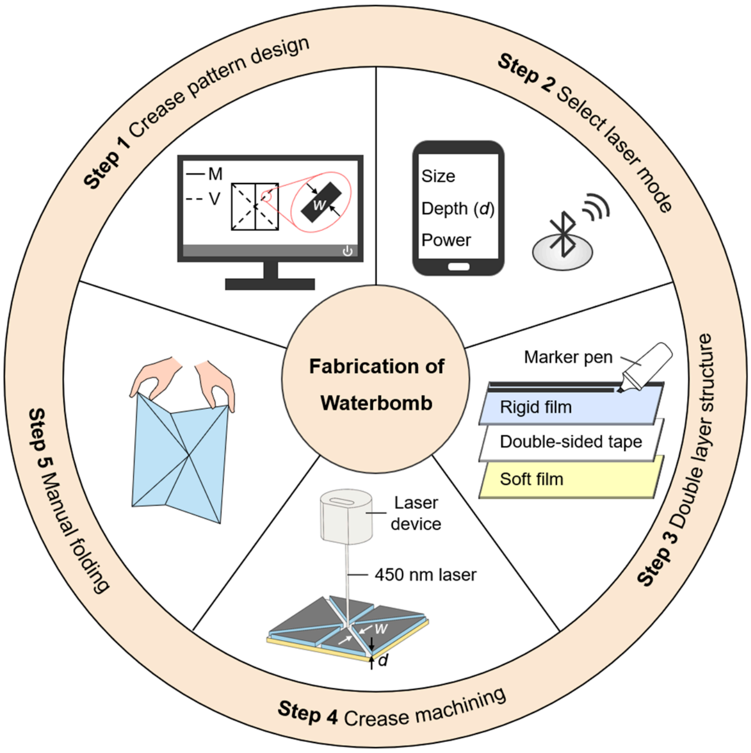

Figure S1. Fabrication of Waterbomb.

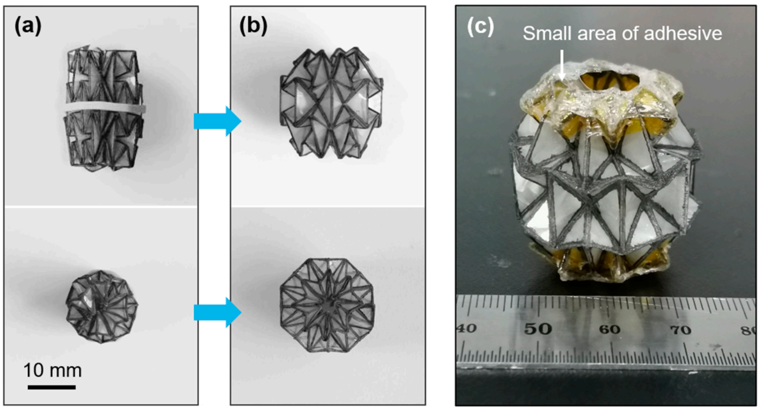

Figure S2. Small size sample of Waterbomb. (**a**) #1. (**b**) #2. (**c**) The seal structure of small size is difficult to bond.

4 Kinematic algorithm diagram

The algorithm analysis steps are as follows, and the detailed diagram is shown in Figure S3.

Step 1, enter the parameter values, including the side length a, the columns n, the angle θ_0.

Step 2, the motion equations are determined by the motion path of P and Q lines.

Step 3, the geometric constraint equations are determined by the connection crease between P and Q lines.

Step 4, if the equations in step 2 and step 3 are compatible with each other, the kinematics

solution results can be output: $Lr = \max\{2r_{P,C}, 2r_{Q,B}\}$, $La = \max\{2z_{P,P}, 2z_{Q,Q}\}$. Otherwise, check the correctness of Step 2 and Step 3.

Step 5, end.

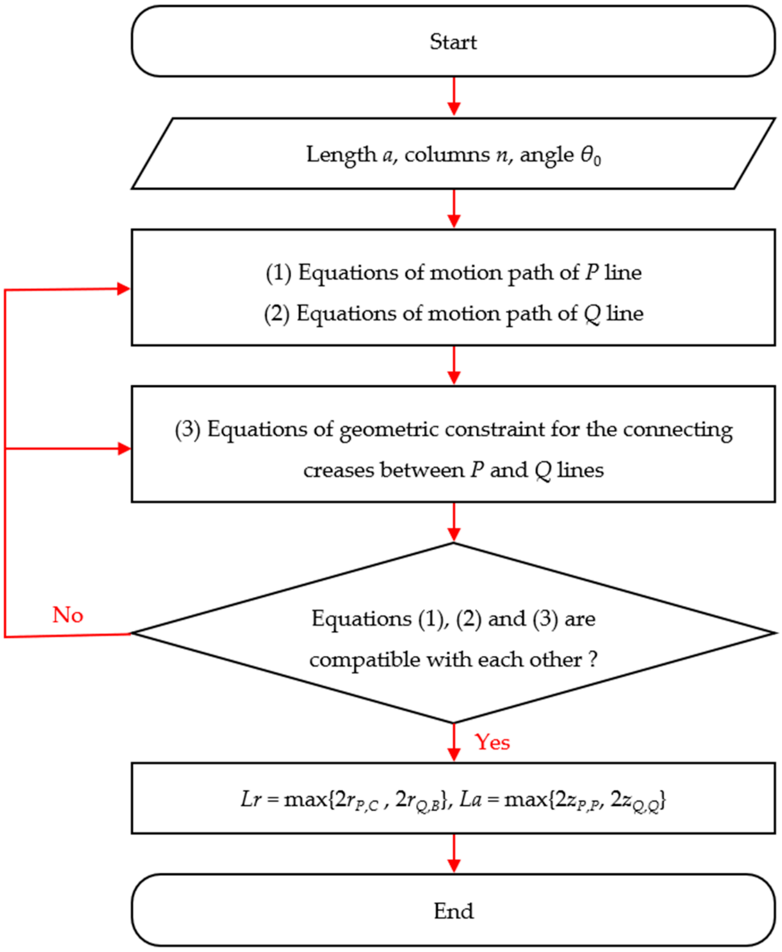

Figure S3. The Waterbomb kinematic algorithm.

5 Vertex classification

In order to describe the motion process of Waterbomb more clearly, we classified all crease intersections and used i and j to locate them, as shown in Figure S4. As can be seen, the Waterbomb consists of three types of inner six-crease vertices and two types of edge vertices. Due to the symmetry of the Waterbomb CPs, we only need to select half columns for analysis.

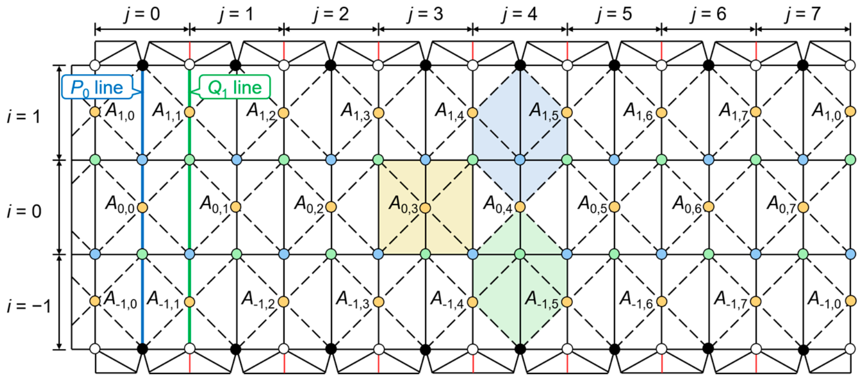

Figure S4. Complete CPs of Waterbomb (m = 3, n = 8). Three types of six-crease vertices (solid circles in yellow, green and blue) and two types of edge vertices (solid circles in black and white) are included. In addition, the P and Q lines indicating the left and right mountain creases in one of the columns.

6 Kinematic modeling

We assume that the transformation process from #1 to #2 satisfies symmetric folding, and θ_0 is taken as the only input variable in the kinematic model. To observe the radial deformation trend of Waterbomb more clearly, we define several key parameters: (1) The dihedral angle β_i of the two smallest triangles facets with common mountain creases at the base of row i. (2) The dihedral angle φ_i of the two largest triangles adjacent to the base. (3) The dihedral angle γ_i of the smallest triangle facets. Among them, β_1 represents the geometric constraint of the end cap on the Waterbomb, which remains constant throughout the movement, and this angle determines the difficulty of axial closure, especially for the manufacture of small specifications.

Considering several important folding angles in the radial direction:

$$\theta_{\text{Refl}} = \frac{\pi}{n}, \theta_{0(\text{half})} = \frac{\theta_0}{2}, \beta_{1(\text{half})} = \frac{\beta_1}{2}, \gamma_{0(\text{half})} = \frac{\gamma_0}{2}$$

where γ_0 satisfies the following equation,

$$\gamma_0 = \cos^{-1}\left(\frac{2a^2 - \left(2r_{\text{P,C}} \sin\theta_{\text{Refl}}\right)^2}{2a^2}\right)$$

Taking $A_{0,j}$ of row 0 as the origin of the local coordinate system $\sum_0 : \text{O}_0 - x_0 y_0 z_0$, see Figure S5 (a and b), we have,

$$\overrightarrow{\text{BC}} = \begin{bmatrix} a\cos\theta_{\text{P,CA}} - a\cos\theta_{0(\text{half})} \\ a\sin\theta_{0(\text{half})} \\ a\sin\theta_{\text{P,CA}} - a \end{bmatrix}$$

Because $\left\|\overrightarrow{\text{BC}}\right\| = a$, therefore,

$$\cos\theta_{\text{P,CA}} \cos\theta_{0(\text{half})} + \sin\theta_{\text{P,CA}} = 1 \quad \text{(S1)}$$

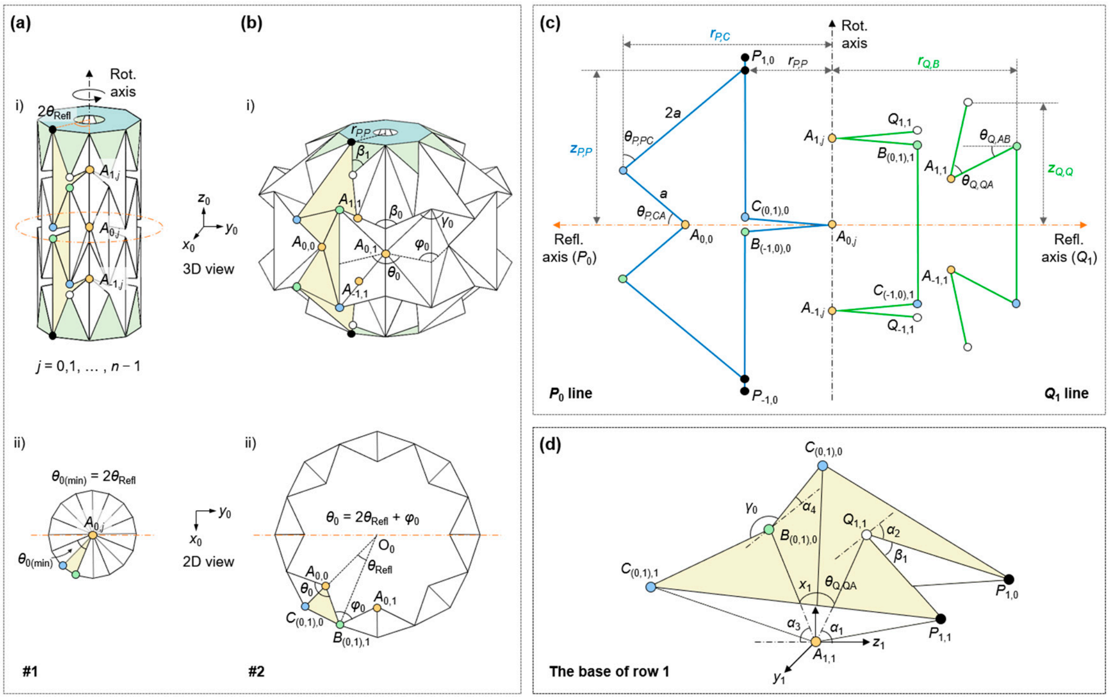

Figure S5. Kinematic analysis of Waterbomb. (**a** and **b**) 3D views of #1 and #2, and 2D projection views of row 0 in the x_0-y_0 plane. (**c**) Motion path of the P and Q lines. (**d**) Position vector model of row 1.

Solve the equation (S1),

$$\theta_{\mathrm{P,CA}} = \sin^{-1}\left(\frac{1-\cos^2\theta_{0(\mathrm{half})}}{1+\cos^2\theta_{0(\mathrm{half})}}\right)$$

$$r_{\mathrm{P,C}} = r_{\mathrm{P,A}} + a\cos\theta_{\mathrm{P,CA}} \tag{S2}$$

When Waterbomb is at #1, $\theta_{0(\mathrm{half})} = \theta_{\mathrm{Refl}}$, then $\theta_{\mathrm{P,CA}}$ has minimum value,

$$\theta_{\mathrm{P,CA(min)}} = \sin^{-1}\left(\frac{1-\cos^2\theta_{\mathrm{Refl}}}{1+\cos^2\theta_{\mathrm{Refl}}}\right)$$

$$r_{\mathrm{P,P}} = a\cos\theta_{\mathrm{P,CA(min)}} \tag{S3}$$

$$\theta_{\mathrm{Q,AB}} = \theta_{\mathrm{P,CA}} - 2\theta_{P,CA(\mathrm{min})}$$

According to the projection view in Figure S5b and the Q line motion path in Figure S5c,

$$r_{\mathrm{Q,B}} = \frac{a\sin\theta_{0(\mathrm{half})}}{\sin\theta_{\mathrm{Refl}}} \tag{S4}$$

$$z_{\mathrm{Q,B}} = a \tag{S5}$$

$$z_{\mathrm{Q,A}} = z_{\mathrm{Q,B}} - a\sin\theta_{\mathrm{Q,AB}} \tag{S6}$$

$$r_{\mathrm{Q,A}} = r_{\mathrm{Q,B}} - \frac{z_{\mathrm{Q,B}} - z_{\mathrm{Q,A}}}{\tan\theta_{\mathrm{Q,AB}}} \tag{S7}$$

In the axial direction, the P and Q lines are constrained in their motion by the connecting folds, and for the vertex P, the following geometric constraint equations exist,

$${r_{\mathrm{Q,A}}}^2 + {r_{\mathrm{P,P}}}^2 - 2r_{\mathrm{Q,A}}r_{\mathrm{P,P}}\cos\theta_{\mathrm{Refl}} + \left(z_{\mathrm{Q,A}} - z_{\mathrm{P,P}}\right)^2 = 2a^2$$

$$z_{\mathrm{P,P}} = z_{\mathrm{Q,A}} + \sqrt{2a^2 - {r_{\mathrm{Q,A}}}^2 - {r_{\mathrm{P,P}}}^2 + 2r_{\mathrm{Q,A}}r_{\mathrm{P,P}}\cos\theta_{\mathrm{Refl}}} \tag{S8}$$

Taking $A_{1,j}$ of row 1 as the origin of the local coordinate system $\sum_1 : O_1 - x_1y_1z_1$, see Figure S5d, we have,

$$\overrightarrow{CP} = \begin{bmatrix} a(\sin\alpha_1 - \cos\alpha_1\sin\alpha_2 - \sin\alpha_3 + \cos\alpha_3\sin\alpha_4) \\ -a(\cos\alpha_2 - \cos\alpha_4) \\ a(\cos\alpha_1 + \sin\alpha_1\sin\alpha_2 + \cos\alpha_3 + \sin\alpha_3\sin\alpha_4) \end{bmatrix}$$

Because $\|\overrightarrow{\mathrm{CP}}\| = 2a$, then,

$$(\sin\alpha_2 + \sin\alpha_4)\sin(\alpha_1 + \alpha_3) + (1 - \sin\alpha_2\sin\alpha_4)\cos(\alpha_1 + \alpha_3) - \cos\alpha_2\cos\alpha_4 = 0$$

Therefore,

$$\left(\cos\beta_{1(\mathrm{half})} + \cos\gamma_{0(\mathrm{half})}\right)\sin\theta_{\mathrm{Q,QA}} - \left(1 - \cos\beta_{1(\mathrm{half})}\cos\gamma_{0(\mathrm{half})}\right)\cos\theta_{\mathrm{Q,QA}} - \sin\beta_{1(\mathrm{half})}\sin\gamma_{0(\mathrm{half})} = 0$$

$$z_{\mathrm{Q,Q}} = z_{\mathrm{Q,A}} + a\sin\left(\theta_{\mathrm{Q,AB}} + \theta_{\mathrm{Q,QA}}\right) \quad \text{(S9)}$$

To sum up, all kinematic parameters of the Waterbomb are determined only by the folding angle θ_0, the radial length $Lr = \max\{2r_{P,C}, 2r_{Q,B}\}$ and the axial length $La = \max\{2z_{P,P}, 2z_{Q,Q}\}$. The model was finally solved by MATLAB software and the results are shown in Figure 3d of the manuscript.

7 Stability judgment of Waterbomb deformation process

Waterbomb has three stable configurations at the same time, because the junction of row 0 and row -1/1 have geometric conflict during deformation, resulting in energy barriers between the different configurations.

On the one hand, it should be noted that of four configurations, only #3 is axially asymmetric, while the remaining three configurations (#1, #2, #4) are all axially symmetric. If the Waterbomb is assumed to have an ideal symmetric motion, the asymmetric path (#1→#2→#3→#4) will become a symmetric path (#1→#2→#4). In a symmetric path, row 1 and row -1 can be nested into row 0 synchronously. Since the space of row 0 is finite (height = $2a$), Waterbomb will has geometric conflict when row 1 and row -1 touch each other, which is equivalent to $z_{Q,A} = 0$, as shown in Figure S5c. Therefore, when

$$z_{Q,A} \neq 0, \quad \text{(S10)}$$

Waterbomb has no geometric conflict. When

$$z_{Q,A} = 0, \quad \text{(S11)}$$

Waterbomb has geometric conflict.

On the other hand, it should be noted that the essence of Waterbomb expansion (axial compression) is to replace $r_{P,C} - r_{P,P}$ with virtual edge $2a\sin\theta_{P,PC}$. Theoretically, when Waterbomb is in a non-motion state

$$r_{P,C} - r_{P,P} = 2a\sin\theta_{P,PC}, \quad \text{(S12)}$$

as shown in Figure S5c. However, this equation is not always satisfied during deformation, as shown in Figure S6. The reason is that $r_{P,P}$ is a constant, and $r_{P,C}$ is determined by $\theta_{P,CA}$, but the change ratio of $\theta_{P,CA}$ and $\theta_{P,PC}$ is not equal, resulting in the anomaly of the equation (3). Therefore, when

$$r_{P,C} - r_{P,P} > 2a\sin\theta_{P,PC}, \quad \text{(S13)}$$

Waterbomb has no geometric conflict, and the motion behavior is consistent with rigid origami (facets are not deformed). When

$$r_{P,C} - r_{P,P} < 2a\sin\theta_{P,PC}, \quad \text{(S14)}$$

Waterbomb has geometric conflict, and the motion behavior is consistent with non-rigid origami (facets are deformed). The analysis results discussed above are shown in Table S3.

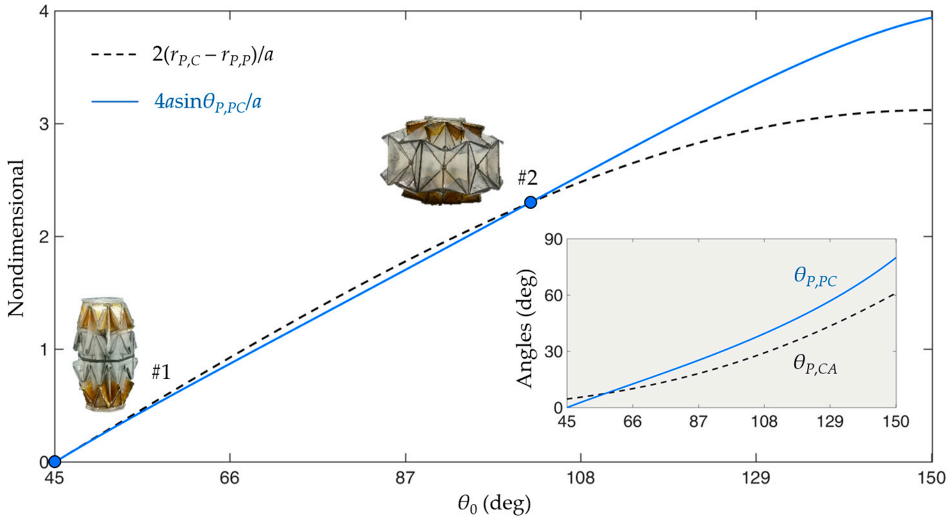

Figure S6. Geometric conflict.

Table S3. Stability judgment of Waterbomb deformation process.

Condition	Geometric conflict	Stability	Motion behavior
$r_{P,C} - r_{P,P} > 2a\sin\theta_{P,PC}$	No	Unstable	Rigid origami
$r_{P,C} - r_{P,P} < 2a\sin\theta_{P,PC}$	Yes	Stable	Non-rigid origami
$z_{Q,A} \neq 0$	No	Unstable	Rigid origami
$z_{Q,A} = 0$	Yes	Stable	Non-rigid origami

8 Stability judgment of various Waterbomb configurations

When Waterbomb is at #1,

$$\theta_{P,PC} = 0, \text{ and } z_{Q,A} \neq 0. \tag{S15}$$

Axial compression of #1 will result in $r_{P,C} - r_{P,P} > 2a\sin\theta_{P,PC}$, so #1 is a unstable configuration. When $r_{P,C} - r_{P,P} = 2a\sin\theta_{P,PC}$, it indicates that #1 has been transformed into #2.

When Waterbomb is at #2,

$$0 < \theta_{P,PC} < \pi/2, \text{ and } z_{Q,A} \neq 0. \tag{S16}$$

Axial compression of #2 will result in $r_{P,C} - r_{P,P} > 2a\sin\theta_{P,PC}$, so #2 is a stable configuration. In order to maintain the stability of #2, Waterbomb will reduce the difference between $r_{P,C} - r_{P,P}$ and $2a\sin\theta_{P,PC}$ by deformation of facets during compression. When $\theta_{P,PC}$ exceed the critical value π/2, it indicates that #2 has been transformed into #3.

When Waterbomb is at #3,

$$\pi/2 < \theta_{P,PC} < \pi. \tag{S17}$$

Since #3 is axially asymmetric, when row 1 (or row -1) satisfies $z_{Q,A} = 0$, axial compression of row -1 (or row 1) on the other side will result in $r_{P,C} - r_{P,P} > 2a\sin\theta_{P,PC}$, so #3 is a stable configuration.

When Waterbomb is at #4,

$$\pi/2 < \theta_{P,PC} < \pi, \text{ and } z_{Q,A} = 0. \tag{S18}$$

As row 1 and row -1 of #4 touch each other, Waterbomb reaches the limit of the axial compression process. According to the symmetry principle and the periodic characteristics of $2a\sin\theta_{P,PC}$, axial stretching of #4 (#4→#2) is equivalent to axial compression of #2 (#2→#4), which will also result in $r_{P,C} - r_{P,P} > 2a\sin\theta_{P,PC}$, so #4 is a stable configuration. If the axial direction continues to compress,

the Waterbomb structure will be destroyed. The analysis results discussed above are shown in Table S4.

Table S4. Stability judgment of various Waterbomb configurations.

**Configuration**	$\theta_{P,PC}$	$z_{Q,A}$	**Axial compression**	**Stability**
#1	0	$z_{Q,A} \neq 0$	$r_{P,C} - r_{P,P} > 2a\sin\theta_{P,PC}$	Unstable
#2	(0, π/2)	$z_{Q,A} \neq 0$	$r_{P,C} - r_{P,P} < 2a\sin\theta_{P,PC}$	Stable
#3	(π/2, π)	$z_{Q,A} = 0$	$r_{P,C} - r_{P,P} < 2a\sin\theta_{P,PC}$	Stable
#4	(π/2, π)	$z_{Q,A} = 0$	$r_{P,C} - r_{P,P} < 2a\sin\theta_{P,PC}$	Stable

References:

1. Masclans, J.R.; Roca, O. High-flow Oxygen Therapy in Acute Respiratory Failure. *Clinical Pulmonary Medicine* **2012**, *19*, 127-130, doi:10.1097/CPM.0b013e3182514f29.
2. Frat, J.P.; Thille, A.W.; Mercat, A.; Girault, C.; Ragot, S.; Perbet, S.; Prat, G.; Boulain, T.; Morawiec, E.; Cottereau, A.; et al. High-flow oxygen through nasal cannula in acute hypoxemic respiratory failure. *N Engl J Med* **2015**, *372*, 2185-2196, doi:10.1056/NEJMoa1503326.
3. Hernandez, G.; Vaquero, C.; Gonzalez, P.; Subira, C.; Frutos-Vivar, F.; Rialp, G.; Laborda, C.; Colinas, L.; Cuena, R.; Fernandez, R. Effect of Postextubation High-Flow Nasal Cannula vs Conventional Oxygen Therapy on Reintubation in Low-Risk Patients: A Randomized Clinical Trial. *JAMA* **2016**, *315*, 1354-1361, doi:10.1001/jama.2016.2711.
4. American Association for Respiratory, C.; Restrepo, R.D.; Walsh, B.K. Humidification during invasive and noninvasive mechanical ventilation: 2012. *Respir Care* **2012**, *57*, 782-788, doi:10.4187/respcare.01766.
5. Hess, D.R. Respiratory mechanics in mechanically ventilated patients. *Respir Care* **2014**, *59*, 1773-1794, doi:10.4187/respcare.03410.
6. Lellouche, F.; Taille, S.; Maggiore, S.M.; Qader, S.; L'Her, E.; Deye, N.; Brochard, L. Influence of ambient and ventilator output temperatures on performance of heated-wire humidifiers. *Am J Respir Crit Care Med* **2004**, *170*, 1073-1079, doi:10.1164/rccm.200309-1245OC.
7. Hill, N.S.; Brennan, J.; Garpestad, E.; Nava, S.J.C.C.M. Non-invasive ventilation in acute respiratory failure. **2002**, *35*, 2402-2407.
8. Pathak, V.; Welsby, I.; Mahmood, K.; Wahidi, M.; MacIntyre, N.; Shofer, S. Ventilation and anesthetic approaches for rigid bronchoscopy. *Ann Am Thorac Soc* **2014**, *11*, 628-634, doi:10.1513/AnnalsATS.201309-302FR.
9. Welham, S.A.; O'Driscoll, B.R.; Howard, L.S.G.; Davison, A.G.; Head, S.E.J.O.-.; Surgery, N. P50–BTS Guideline for Emergency Oxygen Use in Adult Patients. **2010**.
10. Earis; J.; Mak; V.; Howard; L., S.; O'Driscoll; B., R.J.T.T.J.o.t.B.T.S. BTS guideline for oxygen use in adults in healthcare and emergency settings. **2017**.
11. Hanania; Nicola; Sharma; Gulshan; Sharafkhaneh; Respiratory, A.J.S.i.; Medicine, C.C. COPD in the Elderly Patient. **2010**, *31*, 596-606.
12. Donald, T.J.I.J.o.C.O.P.D. A review of nebulized drug delivery in COPD. **2016**, *11*, 2585-2596.
13. Matera, M.G.; Rogliani, P.; Calzetta, L.; Cazzola, M.J.D.S. Safety Considerations with Dual Bronchodilator Therapy in COPD: An Update. **2016**, *39*, 501-508.
